# How to reasonably deal with zero-events in meta-analysis of surgery-related outcomes? Oncologic outcomes of intersphincteric resection vs. abdominoperineal resection for lower rectal cancer: a systematic review and meta-analysis

**DOI:** 10.1097/JS9.0000000000000379

**Published:** 2023-06-07

**Authors:** Rui Zhang, Fei-Long Ning, Chun-Dong Zhang

**Affiliations:** a Department of Colorectal Surgery, Cancer Hospital of China Medical University, Liaoning Cancer Hospital and Institute; b Department of Surgical Oncology, The Fourth Affiliated Hospital of China Medical University; c Department of Gastrointestinal Surgery, The Fourth Affiliated Hospital of China Medical University, Shenyang, People’s Republic of China; d Department of Gastrointestinal Surgery, The University of Tokyo Hospital, Tokyo, Japan

*Dear Editor*,

With great interest, we read a recent systematic review and meta-analysis reported by Du and his colleagues^1^. This systematic review and meta-analysis included a total of 20 nonrandomized controlled studies with 1217 lower rectal cancer patients who underwent intersphincteric resection (ISR) and 1135 patients who underwent abdominoperineal resection (APR). In this study, Du and his colleagues compared ISR with APR in terms of both oncological and surgical safety outcomes. They found that ISR and APR had comparable oncological outcomes, for example, 5-year overall, disease-free, and local recurrence-free survivals. However, the authors did not conduct a meta-analysis of postoperative mortality (POM) between ISR and APR, due to the reason that zero-events of POM were reported in most of the included studies. Therefore, we are still not sure about the surgical safety between these two surgical procedures (i.e. ISR and APR) for patients with lower rectal cancer, regarding that POM (e.g. 30-day or in-hospital mortality) is one of the most important indicators for evaluating surgical safety^2^. We would like to share some opinions.

Meta-analysis is widely applied to synthesize evidence for studies on the same topic. While during the meta-analysis of rare events, for example postoperative mortality, researchers often have difficulties in dealing with zero-events. Notably, Xu *et al*.^3^ have carried out a pioneering study with a five-step principle, to clearly classify meta-analyses with zero-events studies into six subtypes, helping researchers better deal with zero-events in meta-analysis. Based on this pioneering study, the current meta-analysis is considered as a meta-analysis that contains zero events occurring in both single and double arms, and the total event count in neither arm is zero (MA-MZ subtype). For the current meta-analysis, the Mantel–Haenszel (M–H) method measured by risk difference (RD) is preferred. Furthermore, sensitivity analysis of removing all double-arm-zero-events studies can be conducted to test whether the results are robust^3^.

To add new evidence of the surgical safety between ISR and APR, we conducted a further meta-analysis and sensitivity analysis in terms of POM (i.e. 30-day or in-hospital mortality). Fifteen of 20 studies had available data on POM in the meta-analysis. Accordingly, nine studies have double-arm-zero events of POM, and five studies have single-arm-zero-events of POM (Supplementary Table 1, Supplemental Digital Content 1, http://links.lww.com/JS9/A672). There was no significant difference in POM among lower rectal cancer patients undergoing ISR and APR (M–H, fixed model, RD, −0.00; 95% CI, −0.01 to 0.01; *P*=0.95), and no heterogeneity could be observed among the 15 studies (test for heterogeneity, *I* ^2^=0%, *P*=0.89) (Supplementary Fig. 1a, Supplemental Digital Content 2, http://links.lww.com/JS9/A673).

Sensitivity analysis was further conducted by removing nine double-arm-zero-events. Consistently, no significant difference in POM was observed between patients undergoing ISR and those undergoing APR (M–H, fixed model, RD, −0.00; 95% CI, −0.02 to 0.02; *P*=0.94), and there was still no significant heterogeneity among the six studies (test for heterogeneity, *I* ^2^=38%, *P*=0.16) (Supplementary Figure 1b, Supplemental Digital Content 2, http://links.lww.com/JS9/A673). These results suggested that the surgical safety was comparable between ISR and APR surgery for lower rectal cancer patients. These findings may add important new evidence to the current systematic review and meta-analysis conducted by Du and his colleagues^1^.

Dealing with zero-events studies in a meta-analysis inappropriately may cause biased results and even mislead clinical practice. Therefore, we should be cautious to deal with zero-events studies in a meta-analysis of surgery-related outcomes. To make better decisions, it is crucial to select appropriate synthesis methods for meta-analyses. This comprehensive and simple framework proposed by Xu and his colleagues should be preferred for conducting meta-analysis with zero-events studies in surgery-related outcomes.

## Ethical approval and consent to participants

Not applicable.

## Sources of funding

C.-D. Z. was partly supported by the China Scholarship Council (201908050148). R. Z. was partly supported by the National Natural Science Foundation of China (61976249). These funding sources had no role in the study design, in the collection, analysis, and interpretation of data, in the writing, and in the decision to submit the article.

## Author contribution

R.Z. and F.-L.N.: writing and editing; C.-D.Z.: conceptualization, writing, editing, revision, and submission.

## Conflicts of interest disclosure

The authors declare no conflicts of interest.

## Data availability statement

The data supporting the findings of this study are available from the corresponding author upon reasonable request.

## Research registration unique identifying number (UIN)

Not applicable.

## Guarantor

Chun-Dong Zhang.

## Supplementary Material
