# Supplementary material for: How to reasonably deal with zero-events in meta-analysis of surgery-related outcomes? Oncologic outcomes of intersphincteric resection vs. abdominoperineal resection for lower rectal cancer: a systematic review and meta-analysis
Source: Int J Surg. 2023 Jun 7;109(7):2137–8. doi: 10.1097/JS9.0000000000000379 (PMC10389631; doi:10.1097/JS9.0000000000000379)
Supplement: Supplementary file 1 [file js9-109-2137-s001.docx]

**Supplementary Table 1** Clinical characteristics of from 20 included studies.

| **Study** | **Country** | **Data period** | **Intersphincteric resection (ISR)** | | **Abdominoperineal resection (APR)** | | **Definition of POM** |
| --- | --- | --- | --- | --- | --- | --- | --- |
|  |  |  | **POM (*r1*)** | **Total (*n1*)** | **POM (*r2*)** | **Total (*n2*)** |  |
| Akagi Y et al. 2013 ^[1]^ | Japan | 2001–2011 | 0 | 124 | 0 | 60 | In-hospital mortality |
| Beppu N et al. 2017 ^[2]^ | Japan | 2003–2015 | N.A. | 104 | N.A. | 15 | N.A. |
| Braun J et al. 1992 ^[3]^ | Germany | 1977–1987 | 4 | 63 | 0 | 77 | In-hospital mortality |
| Dumont F et al. 2013 ^[4]^ | France | 1995–2011 | 0 | 14 | 0 | 22 | In-hospital mortality |
| Fukuoka H et al. 2020 ^[5]^ | Japan | 2011–2016 | 0 | 17 | 0 | 19 | 30-day mortality |
| He Z et al. 2021 ^[6]^ | China | 2012–2018 | 1 | 43 | 0 | 31 | In-hospital mortality |
| Hohenberger W et al. 2006 ^[7]^ | Germany | 1985–2001 | 2 | 65 | 13 | 285 | In-hospital mortality |
| Kim CH et al. 2015 ^[8]^ | Korea | 2006–2011 | N.A. | 162 | N.A. | 40 | N.A. |
| Klose J et al. 2017 ^[9]^ | Germany | 2001–2012 | 0 | 60 | 0 | 83 | In-hospital mortality |
| Konanz J et al. 2013 ^[10]^ | Germany | 1999–2009 | N.A. | 33 | N.A. | 50 | N.A. |
| Koyama M et al. 2014 ^[11]^ | Japan | 2000–2007 | N.A. | 77 | N.A. | 33 | N.A. |
| Kuo LJ et al. 2011 ^[12]^ | China | 2002–2009 | 1 | 26 | 0 | 23 | In-hospital mortality |
| Miyajima N et al. 2008 ^[13]^ | Japan | 1994–2006 | 0 | 14 | 0 | 14 | 30-day mortality |
| Molnar C et al. 2019 ^[14]^ | Romania | 2011–2013 | N.A. | 37 | N.A. | 35 | N.A. |
| Rubinkiewicz M et al. 2018 ^[15]^ | Poland | 2015–2017 | 0 | 14 | 0 | 13 | 30-day mortality |
| Rullier E et al. 2013 ^[16]^ | France | 1994–2009 | 0 | 186 | 3 | 83 | In-hospital mortality |
| Saito N et al. 2009 ^[17]^ | Japan | 1995–2006 | 0 | 132 | 0 | 70 | In-hospital mortality |
| Shin JK et al. 2022 ^[18]^ | Korea | 2000–2014 | 0 | 104 | 0 | 79 | 30-day mortality |
| Tsukamoto S et al. 2018 ^[19]^ | Japan | 2000–2014 | 0 | 112 | 0 | 173 | In-hospital mortality |
| Weiser MR et al. 2009 ^[20]^ | USA | 1998–2004 | 0 | 44 | 1 | 64 | 30-day mortality |

N.A., not available; POM, postoperative mortality.

**Reference**

1. Akagi Y, Shirouzu K, Ogata Y, *et al*. Oncologic outcomes of intersphincteric resection without preoperative chemoradiotherapy for very low rectal cancer. Surg Oncol 2013;22:144–9.
2. Beppu N, Kimura F, Aihara T, *et al*. Patterns of Local Recurrence and Oncologic Outcomes in T3 Low Rectal Cancer (≤5 cm from the Anal Verge) Treated With Short-Course Radiotherapy With Delayed Surgery : Outcomes in T3 Low Rectal Cancer Treated With Short-Course Radiotherapy With Delayed Surgery. Ann Surg Oncol 2017;24:219–26.
3. Braun J, Treutner KH, Winkeltau G, *et al*. Results of intersphincteric resection of the rectum with direct coloanal anastomosis for rectal carcinoma. Am J Surg 1992;163:407–12.
4. Dumont F, Ayadi M, Goéré D, *et al*. Comparison of fecal continence and quality of life between intersphincteric resection and abdominoperineal resection plus perineal colostomy for ultra-low rectal cancer. J Surg Oncol 2013;108:225–9.
5. Fukuoka H, Fukunaga Y, Minami H, *et al*. Needlescopic surgery for very low rectal cancer with no abdominal skin incision. Asian J Endosc Surg 2020;13:180–5.
6. He Z, Peng B, Chen W, *et al*. Clinical Efficacy of Intersphincteric Resection for Low Rectal Cancer Compared With Abdominoperineal Resection: A Single-Center Retrospective Study. Am Surg 2021:31348211056271.
7. Hohenberger W, Merkel S, Matzel K, *et al*. The influence of abdomino-peranal (intersphincteric) resection of lower third rectal carcinoma on the rates of sphincter preservation and locoregional recurrence. Colorectal Dis 2006;8:23–33.
8. Kim CH, Lee SY, Kim HR, *et al*. Factors Associated With Oncologic Outcomes Following Abdominoperineal or Intersphincteric Resection in Patients Treated With Preoperative Chemoradiotherapy: A Propensity Score Analysis. Medicine (Baltimore) 2015;94:e2060.
9. Klose J, Tarantino I, Kulu Y, *et al*. Sphincter-Preserving Surgery for Low Rectal Cancer: Do We Overshoot the Mark? J Gastrointest Surg 2017;21:885–91.
10. Konanz J, Herrle F, Weiss C, *et al*. Quality of life of patients after low anterior, intersphincteric, and abdominoperineal resection for rectal cancer--a matched-pair analysis. Int J Colorectal Dis 2013;28:679–88.
11. Koyama M, Murata A, Sakamoto Y, *et al*. Long-term clinical and functional results of intersphincteric resection for lower rectal cancer. Ann Surg Oncol 2014;21:S422–8.
12. Kuo LJ, Hung CS, Wu CH, *et al*. Oncological and functional outcomes of intersphincteric resection for low rectal cancer. J Surg Res 2011;170:e93–8.
13. Miyajima N. Short-term outcome of laparoscopic surgery for rectal cancer. Keio J Med 2008;57:150–4.
14. Molnar C, Nicolescu C, Grigorescu BL, *et al*. Comparative oncological outcomes and survival following surgery for low rectal cancer - a single center experience. Rom J Morphol Embryol 2019;60:847–52.
15. Rubinkiewicz M, Zarzycki P, Czerwińska A, *et al*. A quest for sphincter-saving surgery in ultralow rectal tumours-a single-centre cohort study. World J Surg Oncol 2018;16:218.
16. Rullier E, Denost Q, Vendrely V, *et al*. Low rectal cancer: classification and standardization of surgery. Dis Colon Rectum 2013;56:560–7.
17. Saito N, Sugito M, Ito M, *et al*. Oncologic outcome of intersphincteric resection for very low rectal cancer. World J Surg 2009;33:1750–6.
18. Shin JK, Kim HC, Lee WY, *et al*. Sphincter-saving surgery versus abdominoperineal resection in low rectal cancer following neoadjuvant treatment with propensity score analysis. Surg Endosc 2022;36:2623–30.
19. Tsukamoto S, Miyake M, Shida D, *et al*. Intersphincteric Resection Has Similar Long-term Oncologic Outcomes Compared With Abdominoperineal Resection for Low Rectal Cancer Without Preoperative Therapy: Results of Propensity Score Analyses. Dis Colon Rectum 2018;61:1035–42.
20. Weiser MR, Quah HM, Shia J, *et al*. Sphincter preservation in low rectal cancer is facilitated by preoperative chemoradiation and intersphincteric dissection. Ann Surg 2009;249:236–42.
