# Supplementary figures and images for: How to reasonably deal with zero-events in meta-analysis of surgery-related outcomes? Oncologic outcomes of intersphincteric resection vs. abdominoperineal resection for lower rectal cancer: a systematic review and meta-analysis
Source: Int J Surg. 2023 Jun 7;109(7):2137–8. doi: 10.1097/JS9.0000000000000379 (PMC10389631; doi:10.1097/JS9.0000000000000379)

a

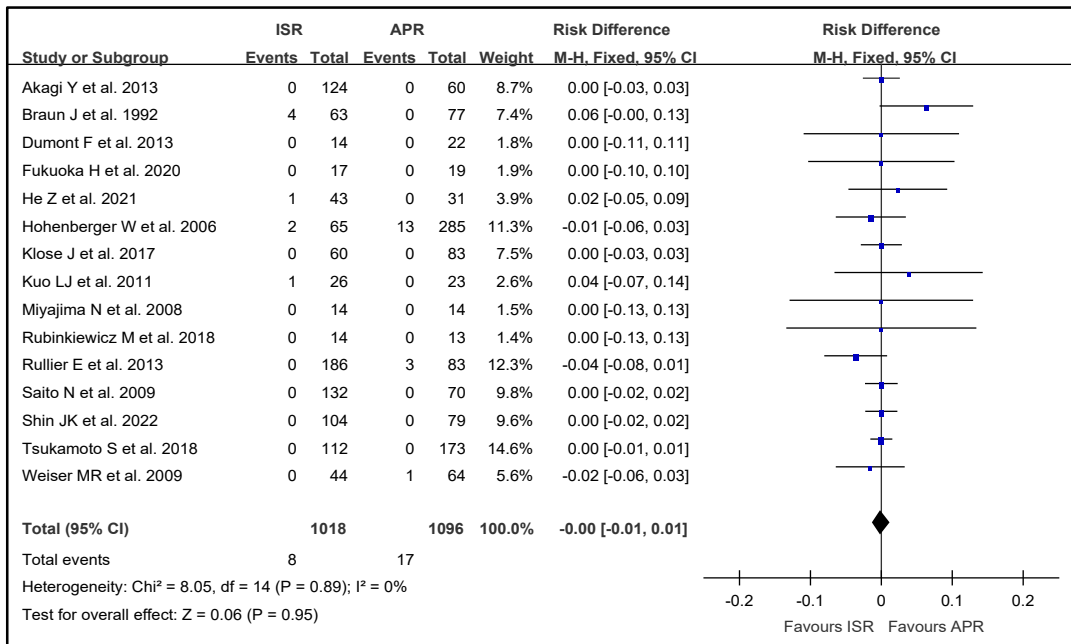

b

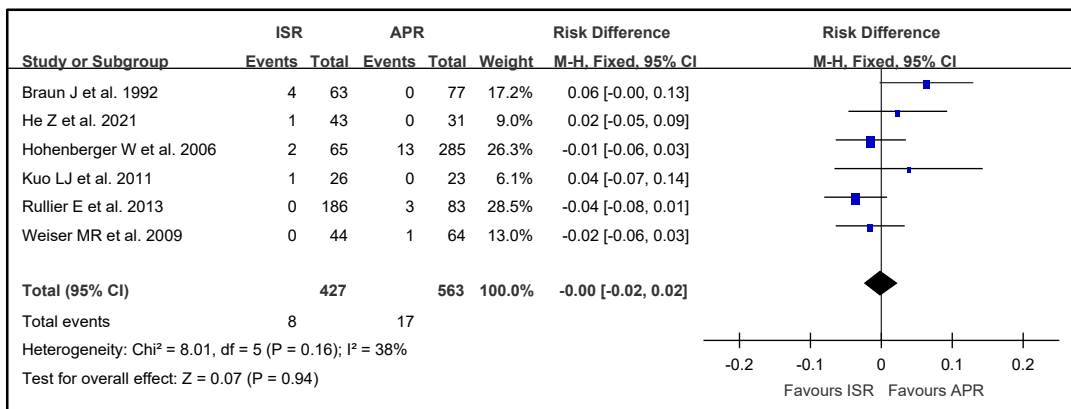

Supplementary Figure 1

Supplement: Supplementary file 2 [file js9-109-2137-s002.pdf]
